# (1*E*,4*Z*,6*E*)-5-Hy­droxy-1,7-bis­(2-meth­oxy­phen­yl)-1,4,6-hepta­trien-3-one

**DOI:** 10.1107/S160053681102469X

**Published:** 2011-07-02

**Authors:** Yiliang Zhao, Paul W. Groundwater, David E. Hibbs, Paul K. Nguyen, Rajeshwar Narlawar

**Affiliations:** a Faculty of Pharmacy, The University of Sydney, NSW 2006, Australia

## Abstract

In the title compound, C_21_H_20_O_4_, the central hepta­trienone unit is approximately planar, with a maximum atomic deviation of 0.1121 (11) Å; the two benzene rings are twisted with respect to the hepta­trienone mean plane by 2.73 (5) and 29.31 (4)°. The mol­ecule exists in the enol form and the hy­droxy group forms an intra­molecular hydrogen bond with the neighboring carbonyl group. Weak inter­molecular C—H⋯O hydrogen bonding is present in the crystal structure.

## Related literature

For potential applications of curcumin and its derivatives in medicine, see: Reddy & Lokesh (1992 ▶); Sreejayan Rao (1997 ▶); Narlawar *et al.* (2008 ▶); Qiu *et al.* (2010 ▶). For the tautomerism of curcumin and its analogues, see: Gunasekaran *et al.* (2008 ▶).
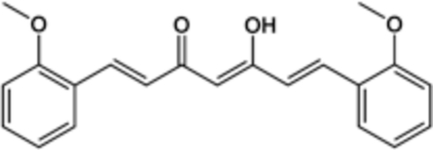


## Experimental

### 

#### Crystal data

C_21_H_20_O_4_
*M* *_r_* = 336.37
Triclinic, 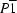

*a* = 7.3234 (11) Å
*b* = 7.7960 (12) Å
*c* = 16.897 (3) Å
α = 96.819 (3)°
β = 95.641 (3)°
γ = 115.520 (2)°
*V* = 852.3 (2) Å^3^
*Z* = 2
Mo *K*α radiation
μ = 0.09 mm^−1^
*T* = 150 K
0.30 × 0.20 × 0.20 mm

#### Data collection

Bruker SMART APEXII CCD diffractometer
5805 measured reflections
2998 independent reflections
2238 reflections with *I* > 2σ(*I*)
*R*_int_ = 0.022

#### Refinement

*R*[*F* ^2^ > 2σ(*F* ^2^)] = 0.044
*wR*(*F* ^2^) = 0.120
*S* = 1.05
2998 reflections
229 parameters
H-atom parameters constrained
Δρ_max_ = 0.19 e Å^−3^
Δρ_min_ = −0.24 e Å^−3^

### 

Data collection: *APEX2* (Bruker, 2008 ▶); cell refinement: *SAINT* (Bruker, 2008 ▶); data reduction: *SAINT*; program(s) used to solve structure: *SHELXS97* (Sheldrick, 2008 ▶); program(s) used to refine structure: *SHELXL97* (Sheldrick, 2008 ▶); molecular graphics: *ORTEP-3 for Windows* (Farrugia, 1999 ▶); software used to prepare material for publication: *SHELXL97* and *publCIF* (Westrip, 2010 ▶).

## Supplementary Material

Crystal structure: contains datablock(s) global, I. DOI: 10.1107/S160053681102469X/xu5219sup1.cif

Structure factors: contains datablock(s) I. DOI: 10.1107/S160053681102469X/xu5219Isup2.hkl

Supplementary material file. DOI: 10.1107/S160053681102469X/xu5219Isup3.cml

Additional supplementary materials:  crystallographic information; 3D view; checkCIF report

## Figures and Tables

**Table 1:** Hydrogen-bond geometry (Å, °)

*D*—H⋯*A*	*D*—H	H⋯*A*	*D*⋯*A*	*D*—H⋯*A*
O3—H3′⋯O2	0.82	1.77	2.5003 (18)	147
C5—H5⋯O2^i^	0.93	2.45	3.351 (2)	162
C8—H8⋯O2^i^	0.93	2.49	3.413 (2)	169

Symmetry code: (i) 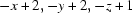.

**Fig. 1.**
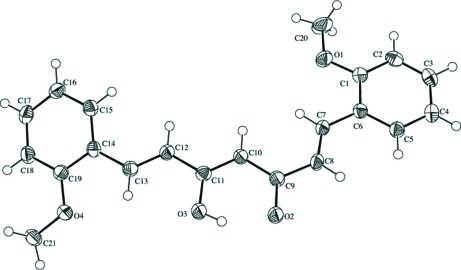
The molecular structure of the title compound with 50% probability displacement ellipsoids for non-H atoms.

## supplementary crystallographic information

### Comment

The title compound is a derivative of curcumin. The curcumin and its derivatives have potent applications in the medicine field (Reddy & Lokesh, 1992; Sreejayan Rao, 1997; Narlawar *et al.*, 2008; Qiu *et al.*, 2010). We synthesized non-natural curcumin analogs in order to increase the NF-κ*B* inhibitory activity and reduce the cell toxicity.

The molecular structure of the compound is showing in Fig. 1. The bond lengths indicate the electron delocalization in the molecular structure. The central heptatrienone unit is approximately planar with the maximum atomic deviation of -0.1121 (11) Å; the two benzene rings are twisted with respect to the heptatrienone mean plane at 2.73 (5) and 29.31 (4)°, respectively. In the solution, curcumin and its non-natural analogs exist as ketol-enol tautomeric forms (Gunasekaran *et al.*, 2008). In the crystal, the title compound exists as an enol form, the hydroxy group forms an intra-molecular hydrogen bond to the neighboring carbonyl group.

Weak intermolecular C—H···O hydrogen bonding is present in the crystal structure (Table 1).

### Experimental

In a dry three-necked flask, 1.03 ml acetylacetone (10 mmol) and 0.488 g boron oxide (3.5 mmol) were dissolved in 10 ml e thyl acetate and heated to 75°C for 1 h. Methoxybenzaldehyde (2.84 ml, 20 mmol) and tributylborate (4.8 ml, 20 mmol) were mixed with 10 ml e thyl acetate, stirred for 45 min and then added to the solution.

The mixture was heated to 100°C for 1 h, then n-butylamine (1.54 ml, 15 mmol) dissolved in 15 ml e thyl acetate was added dropwise over a period of 90 min. The reaction was stirred for 18 h at 85°C, cooled to 60°C, then 4*M* HCl solution (5 ml) was added and the mixture stirred at 60°C for 1 h.

The reaction mixture was cooled to ambient temperature, the organic layer was separated and the aqueous layer was extracted with ethyl acetate (3 × 100 ml). The combined organic layers were washed with 100 ml of brine, dried over anhydrous Na_2_SO_4_ and evaporated *in vacuo*.

The crude compound was purified by flash column chromatography, using 4% ethyl acetate in petroleum ether to give the title compound as a yellow solid (0.35 g, 5%). Single crystals suitable for X-ray data collection were obtained by slow evaporation of an ethyl acetate:petroleum ether (2:8) solution.

### Refinement

H atoms were placed in calculated positions with O—H = 0.82, C—H = 0.93 to 0.96 Å, and were refined in a riding mode with *U*_iso_(H) = 1.2*U*_eq_(C) for aromatic H atoms and 1.5*U*_eq_(C,*O*) for the others.

### Crystal data

**Table:**

C_21_H_20_O_4_	*Z* = 2
*M**_r_* = 336.37	*F*(000) = 356
Triclinic, *P*1	*D*_x_ = 1.311 Mg m^−^^3^
Hall symbol: -P 1	Melting point: 397(2) K
*a* = 7.3234 (11) Å	Mo *K*α radiation, λ = 0.71073 Å
*b* = 7.7960 (12) Å	Cell parameters from 500 reflections
*c* = 16.897 (3) Å	θ = 1.1–28.2°
α = 96.819 (3)°	µ = 0.09 mm^−^^1^
β = 95.641 (3)°	*T* = 150 K
γ = 115.520 (2)°	Block, yellow
*V* = 852.3 (2) Å^3^	0.30 × 0.20 × 0.20 mm

### Data collection

**Table:**

Bruker SMART APEXII CCD diffractometer	2238 reflections with *I* > 2σ(*I*)
Radiation source: fine-focus sealed tube, Bruker K760	*R*_int_ = 0.022
graphite	θ_max_ = 25.2°, θ_min_ = 2.5°
ω scans	*h* = −8→8
5805 measured reflections	*k* = −7→9
2998 independent reflections	*l* = −20→20

### Refinement

**Table:**

Refinement on *F*^2^	Primary atom site location: structure-invariant direct methods
Least-squares matrix: full	Secondary atom site location: difference Fourier map
*R*[*F*^2^ > 2σ(*F*^2^)] = 0.044	Hydrogen site location: inferred from neighbouring sites
*wR*(*F*^2^) = 0.120	H-atom parameters constrained
*S* = 1.05	*w* = 1/[σ^2^(*F*_o_^2^) + (0.0634*P*)^2^ + 0.0684*P*] where *P* = (*F*_o_^2^ + 2*F*_c_^2^)/3
2998 reflections	(Δ/σ)_max_ = 0.009
229 parameters	Δρ_max_ = 0.19 e Å^−^^3^
0 restraints	Δρ_min_ = −0.24 e Å^−^^3^

### Special details

**Table:**

Geometry. All e.s.d.'s (except the e.s.d. in the dihedral angle between two l.s. planes) are estimated using the full covariance matrix. The cell e.s.d.'s are taken into account individually in the estimation of e.s.d.'s in distances, angles and torsion angles; correlations between e.s.d.'s in cell parameters are only used when they are defined by crystal symmetry. An approximate (isotropic) treatment of cell e.s.d.'s is used for estimating e.s.d.'s involving l.s. planes.
Refinement. Refinement of *F*^2^ against ALL reflections. The weighted *R*-factor *wR* and goodness of fit *S* are based on *F*^2^, conventional *R*-factors *R* are based on *F*, with *F* set to zero for negative *F*^2^. The threshold expression of *F*^2^ > σ(*F*^2^) is used only for calculating *R*-factors(gt) *etc*. and is not relevant to the choice of reflections for refinement. *R*-factors based on *F*^2^ are statistically about twice as large as those based on *F*, and *R*- factors based on ALL data will be even larger.

### Fractional atomic coordinates and isotropic or equivalent isotropic displacement parameters (Å^2^)

**Table:**

	*x*	*y*	*z*	*U*_iso_*/*U*_eq_	
C1	0.6037 (2)	0.3920 (3)	0.64590 (10)	0.0297 (4)	
C2	0.5514 (3)	0.3937 (3)	0.72270 (10)	0.0344 (4)	
H2	0.4929	0.2793	0.7428	0.041*	
C3	0.5873 (3)	0.5672 (3)	0.76908 (10)	0.0354 (5)	
H3	0.5524	0.5687	0.8205	0.042*	
C4	0.6743 (3)	0.7384 (3)	0.74013 (10)	0.0338 (4)	
H4	0.6964	0.8541	0.7716	0.041*	
C5	0.7279 (2)	0.7363 (3)	0.66415 (9)	0.0297 (4)	
H5	0.7877	0.8518	0.6450	0.036*	
C6	0.6946 (2)	0.5643 (2)	0.61529 (9)	0.0268 (4)	
C7	0.7506 (2)	0.5582 (2)	0.53473 (9)	0.0281 (4)	
H7	0.7175	0.4365	0.5058	0.034*	
C8	0.8438 (2)	0.7075 (3)	0.49809 (10)	0.0304 (4)	
H8	0.8820	0.8307	0.5266	0.036*	
C9	0.8905 (2)	0.6913 (3)	0.41590 (9)	0.0280 (4)	
C10	0.8584 (2)	0.5123 (2)	0.37012 (9)	0.0272 (4)	
H10	0.8122	0.4023	0.3937	0.033*	
C11	0.8942 (2)	0.4988 (2)	0.29240 (9)	0.0268 (4)	
C12	0.8535 (2)	0.3168 (2)	0.24370 (9)	0.0283 (4)	
H12	0.8155	0.2092	0.2687	0.034*	
C13	0.8668 (2)	0.2926 (3)	0.16534 (9)	0.0281 (4)	
H13	0.9086	0.4020	0.1415	0.034*	
C14	0.8221 (2)	0.1110 (2)	0.11348 (9)	0.0264 (4)	
C15	0.8139 (2)	−0.0505 (2)	0.14426 (10)	0.0293 (4)	
H15	0.8443	−0.0406	0.1999	0.035*	
C16	0.7621 (2)	−0.2245 (3)	0.09447 (10)	0.0315 (4)	
H16	0.7570	−0.3302	0.1163	0.038*	
C17	0.7178 (3)	−0.2390 (3)	0.01173 (10)	0.0348 (4)	
H17	0.6811	−0.3559	−0.0221	0.042*	
C18	0.7274 (3)	−0.0814 (3)	−0.02129 (10)	0.0335 (4)	
H18	0.6975	−0.0930	−0.0770	0.040*	
C19	0.7815 (2)	0.0937 (2)	0.02861 (9)	0.0275 (4)	
C20	0.4696 (3)	0.0470 (3)	0.62014 (13)	0.0528 (6)	
H20A	0.5460	0.0455	0.6694	0.079*	
H20B	0.4574	−0.0550	0.5788	0.079*	
H20C	0.3353	0.0284	0.6290	0.079*	
C21	0.7528 (3)	0.2469 (3)	−0.08326 (9)	0.0361 (4)	
H21A	0.8425	0.2087	−0.1101	0.054*	
H21B	0.7722	0.3714	−0.0938	0.054*	
H21C	0.6129	0.1539	−0.1030	0.054*	
O1	0.57382 (19)	0.22818 (18)	0.59524 (7)	0.0404 (3)	
O2	0.95885 (19)	0.84396 (17)	0.38537 (7)	0.0373 (3)	
O3	0.96478 (19)	0.65274 (18)	0.25677 (7)	0.0355 (3)	
H3'	0.9801	0.7482	0.2883	0.053*	
O4	0.79859 (17)	0.25673 (17)	0.00178 (6)	0.0335 (3)	

### Atomic displacement parameters (Å^2^)

**Table:**

	*U*^11^	*U*^22^	*U*^33^	*U*^12^	*U*^13^	*U*^23^
C1	0.0299 (9)	0.0292 (10)	0.0291 (9)	0.0131 (8)	0.0038 (7)	0.0041 (8)
C2	0.0337 (10)	0.0361 (11)	0.0333 (10)	0.0132 (8)	0.0080 (7)	0.0138 (8)
C3	0.0332 (9)	0.0475 (13)	0.0250 (9)	0.0171 (9)	0.0072 (7)	0.0067 (8)
C4	0.0335 (9)	0.0356 (11)	0.0288 (9)	0.0142 (8)	0.0035 (7)	−0.0010 (8)
C5	0.0314 (9)	0.0268 (10)	0.0273 (9)	0.0102 (8)	0.0047 (7)	0.0027 (7)
C6	0.0269 (8)	0.0258 (10)	0.0261 (9)	0.0108 (7)	0.0030 (7)	0.0040 (7)
C7	0.0315 (9)	0.0241 (10)	0.0258 (9)	0.0110 (8)	0.0033 (7)	0.0003 (7)
C8	0.0383 (10)	0.0222 (10)	0.0258 (9)	0.0102 (8)	0.0059 (7)	−0.0008 (7)
C9	0.0288 (9)	0.0245 (10)	0.0270 (9)	0.0085 (7)	0.0045 (7)	0.0046 (7)
C10	0.0312 (9)	0.0234 (9)	0.0245 (8)	0.0100 (7)	0.0046 (7)	0.0042 (7)
C11	0.0262 (8)	0.0236 (9)	0.0282 (9)	0.0091 (7)	0.0032 (7)	0.0052 (7)
C12	0.0313 (9)	0.0241 (10)	0.0287 (9)	0.0113 (8)	0.0066 (7)	0.0049 (7)
C13	0.0287 (9)	0.0260 (10)	0.0283 (9)	0.0107 (7)	0.0063 (7)	0.0052 (7)
C14	0.0243 (8)	0.0281 (10)	0.0264 (9)	0.0113 (7)	0.0065 (6)	0.0035 (7)
C15	0.0287 (9)	0.0302 (10)	0.0283 (9)	0.0122 (8)	0.0067 (7)	0.0048 (7)
C16	0.0328 (9)	0.0268 (10)	0.0371 (10)	0.0143 (8)	0.0099 (7)	0.0066 (8)
C17	0.0365 (10)	0.0283 (11)	0.0361 (10)	0.0133 (8)	0.0079 (8)	−0.0028 (8)
C18	0.0383 (10)	0.0351 (11)	0.0254 (9)	0.0157 (8)	0.0063 (7)	0.0005 (8)
C19	0.0262 (8)	0.0263 (10)	0.0299 (9)	0.0109 (7)	0.0076 (7)	0.0061 (7)
C20	0.0698 (14)	0.0275 (12)	0.0646 (14)	0.0198 (11)	0.0241 (11)	0.0186 (10)
C21	0.0405 (10)	0.0443 (12)	0.0284 (9)	0.0212 (9)	0.0099 (8)	0.0125 (8)
O1	0.0556 (8)	0.0249 (7)	0.0414 (7)	0.0157 (6)	0.0178 (6)	0.0098 (6)
O2	0.0537 (8)	0.0227 (7)	0.0312 (7)	0.0114 (6)	0.0141 (6)	0.0051 (5)
O3	0.0508 (8)	0.0248 (7)	0.0288 (6)	0.0138 (6)	0.0124 (6)	0.0052 (5)
O4	0.0454 (7)	0.0302 (7)	0.0265 (6)	0.0177 (6)	0.0079 (5)	0.0065 (5)

### Geometric parameters (Å, °)

**Table:**

C1—O1	1.369 (2)	C12—H12	0.9300
C1—C2	1.389 (2)	C13—C14	1.460 (2)
C1—C6	1.406 (2)	C13—H13	0.9300
C2—C3	1.384 (3)	C14—C15	1.398 (2)
C2—H2	0.9300	C14—C19	1.414 (2)
C3—C4	1.382 (3)	C15—C16	1.384 (2)
C3—H3	0.9300	C15—H15	0.9300
C4—C5	1.380 (2)	C16—C17	1.384 (2)
C4—H4	0.9300	C16—H16	0.9300
C5—C6	1.398 (2)	C17—C18	1.386 (2)
C5—H5	0.9300	C17—H17	0.9300
C6—C7	1.461 (2)	C18—C19	1.388 (3)
C7—C8	1.332 (2)	C18—H18	0.9300
C7—H7	0.9300	C19—O4	1.359 (2)
C8—C9	1.466 (2)	C20—O1	1.425 (2)
C8—H8	0.9300	C20—H20A	0.9600
C9—O2	1.271 (2)	C20—H20B	0.9600
C9—C10	1.424 (2)	C20—H20C	0.9600
C10—C11	1.365 (2)	C21—O4	1.4283 (18)
C10—H10	0.9300	C21—H21A	0.9600
C11—O3	1.3297 (19)	C21—H21B	0.9600
C11—C12	1.445 (2)	C21—H21C	0.9600
C12—C13	1.334 (2)	O3—H3'	0.8200
			
O1—C1—C2	123.87 (16)	C12—C13—C14	126.25 (16)
O1—C1—C6	115.31 (14)	C12—C13—H13	116.9
C2—C1—C6	120.82 (16)	C14—C13—H13	116.9
C3—C2—C1	119.36 (17)	C15—C14—C19	117.82 (15)
C3—C2—H2	120.3	C15—C14—C13	122.64 (14)
C1—C2—H2	120.3	C19—C14—C13	119.53 (15)
C4—C3—C2	121.01 (16)	C16—C15—C14	121.97 (15)
C4—C3—H3	119.5	C16—C15—H15	119.0
C2—C3—H3	119.5	C14—C15—H15	119.0
C5—C4—C3	119.41 (17)	C17—C16—C15	119.04 (16)
C5—C4—H4	120.3	C17—C16—H16	120.5
C3—C4—H4	120.3	C15—C16—H16	120.5
C4—C5—C6	121.47 (16)	C16—C17—C18	120.78 (17)
C4—C5—H5	119.3	C16—C17—H17	119.6
C6—C5—H5	119.3	C18—C17—H17	119.6
C5—C6—C1	117.92 (15)	C17—C18—C19	120.17 (16)
C5—C6—C7	122.55 (15)	C17—C18—H18	119.9
C1—C6—C7	119.53 (15)	C19—C18—H18	119.9
C8—C7—C6	127.18 (16)	O4—C19—C18	124.39 (15)
C8—C7—H7	116.4	O4—C19—C14	115.44 (14)
C6—C7—H7	116.4	C18—C19—C14	120.18 (16)
C7—C8—C9	124.57 (16)	O1—C20—H20A	109.5
C7—C8—H8	117.7	O1—C20—H20B	109.5
C9—C8—H8	117.7	H20A—C20—H20B	109.5
O2—C9—C10	120.10 (14)	O1—C20—H20C	109.5
O2—C9—C8	117.60 (15)	H20A—C20—H20C	109.5
C10—C9—C8	122.29 (15)	H20B—C20—H20C	109.5
C11—C10—C9	121.33 (15)	O4—C21—H21A	109.5
C11—C10—H10	119.3	O4—C21—H21B	109.5
C9—C10—H10	119.3	H21A—C21—H21B	109.5
O3—C11—C10	121.55 (15)	O4—C21—H21C	109.5
O3—C11—C12	116.30 (14)	H21A—C21—H21C	109.5
C10—C11—C12	122.14 (15)	H21B—C21—H21C	109.5
C13—C12—C11	124.44 (16)	C1—O1—C20	118.23 (14)
C13—C12—H12	117.8	C11—O3—H3'	109.5
C11—C12—H12	117.8	C19—O4—C21	118.11 (13)
			
O1—C1—C2—C3	−179.73 (15)	O3—C11—C12—C13	−5.5 (2)
C6—C1—C2—C3	0.8 (2)	C10—C11—C12—C13	173.19 (15)
C1—C2—C3—C4	0.0 (3)	C11—C12—C13—C14	−178.32 (15)
C2—C3—C4—C5	−0.7 (2)	C12—C13—C14—C15	−18.2 (3)
C3—C4—C5—C6	0.6 (2)	C12—C13—C14—C19	160.64 (15)
C4—C5—C6—C1	0.1 (2)	C19—C14—C15—C16	−1.9 (2)
C4—C5—C6—C7	−179.81 (14)	C13—C14—C15—C16	176.95 (14)
O1—C1—C6—C5	179.67 (14)	C14—C15—C16—C17	0.3 (2)
C2—C1—C6—C5	−0.8 (2)	C15—C16—C17—C18	0.8 (2)
O1—C1—C6—C7	−0.4 (2)	C16—C17—C18—C19	−0.1 (3)
C2—C1—C6—C7	179.11 (15)	C17—C18—C19—O4	178.77 (14)
C5—C6—C7—C8	2.5 (3)	C17—C18—C19—C14	−1.6 (2)
C1—C6—C7—C8	−177.40 (16)	C15—C14—C19—O4	−177.76 (13)
C6—C7—C8—C9	−178.05 (15)	C13—C14—C19—O4	3.3 (2)
C7—C8—C9—O2	172.23 (16)	C15—C14—C19—C18	2.6 (2)
C7—C8—C9—C10	−6.7 (3)	C13—C14—C19—C18	−176.36 (15)
O2—C9—C10—C11	−1.9 (2)	C2—C1—O1—C20	4.7 (2)
C8—C9—C10—C11	177.04 (14)	C6—C1—O1—C20	−175.74 (15)
C9—C10—C11—O3	1.5 (2)	C18—C19—O4—C21	1.7 (2)
C9—C10—C11—C12	−177.04 (14)	C14—C19—O4—C21	−177.93 (13)

### Hydrogen-bond geometry (Å, °)

**Table:**

*D*—H···*A*	*D*—H	H···*A*	*D*···*A*	*D*—H···*A*
O3—H3'···O2	0.82	1.77	2.5003 (18)	147
C5—H5···O2^i^	0.93	2.45	3.351 (2)	162
C8—H8···O2^i^	0.93	2.49	3.413 (2)	169

Symmetry codes: (i) −*x*+2, −*y*+2, −*z*+1.
